# Cochlin, Intraocular Pressure Regulation and Mechanosensing

**DOI:** 10.1371/journal.pone.0034309

**Published:** 2012-04-04

**Authors:** Manik Goel, Adam E. Sienkiewicz, Renata Picciani, Jianhua Wang, Richard K. Lee, Sanjoy K. Bhattacharya

**Affiliations:** Bascom Palmer Eye Institute, University of Miami, Miami, Florida, United States of America; Dalhousie University, Canada

## Abstract

Fluid shear modulates many biological properties. How shear mechanosensing occurs in the extracellular matrix (ECM) and is transduced into cytoskeletal change remains unknown. Cochlin is an ECM protein of unknown function. Our investigation using a comprehensive spectrum of cutting-edge techniques has resulted in following major findings: (1) over-expression and down-regulation of cochlin increase and decrease intraocular pressure (IOP), respectively. The overexpression was achieved in DBA/2J-Gpnmb^+^/SjJ using lentiviral vectors, down-regulation was achieved in glaucomatous DBA/2J mice using targeted disruption (cochlin-null mice) and also using lentiviral vector mediated shRNA against cochlin coding region; (2) reintroduction of cochlin in cochlin-null mice increases IOP; (3) injection of exogenous cochlin also increased IOP; (4) increasing perfusion rates increased cochlin multimerization, which reduced the rate of cochlin proteolysis by trypsin and proteinase K; The cochlin multimerization in response to shear stress suggests its potential mechanosensing. Taken together with previous studies, we show cochlin is involved in regulation of intraocular pressure in DBA/2J potentially through mechanosensing of the shear stress.

## Introduction

Fluid shear is a mechanical stimulus experienced by cells and most organs involved with localized fluid flow. Cellular mechanosensing is linked to cytoskeletal remodeling to respond appropriately to altered fluid shear dynamics in single and multicellular organisms [1].

Fluid flow abnormalities are associated with complex, late onset progressive diseases such as glaucoma (aqueous outflow), idiopathic intracranial hypertension (flow changes in cerebrospinal fluid) and the non-syndromic hearing disorder DFNA9 [2], [3]. IOP fluctuations likely alter cells of the trabecular meshwork (TM), a filter like structure in the anterior eye chamber, results in aqueous outflow dysregulation [4], [5]. Cyclic stretch significantly alters TM gene expression [6]. Regulation of ECM interstitial space is a major influencing factor for aqueous outflow resistance through the trabecular meshwork [7]. Consequently the existence of a mechanosensor (or multiple mechanosensors) to detect fluid shear changes in the ECM is highly plausible. Cochlin, a secreted protein of unknown function was identified in glaucomatous but not normal TM by mass spectrometry and shown to be responsive to fluid shear [8]. Stretch activated channels (SACs), such as TREK-1, function as mechanotransducers involved in pressure regulation [1], [9]. Although TREK-1 mRNA is present in the TM, its role in pressure regulation has not been demonstrated nor has the involvement of cochlin in mechanosensing. Cochlin expression have been previously shown to result in co-expression of TREK-1 and filopodia formation [10]. A direct or functional interaction of cochlin and TREK-1 remains to be demonstrated, however, a potential functional interaction between cochlin and TREK-1 may exist [10]. We provide evidence here that cochlin is involved in IOP regulation.

## Results

### Cochlin Mediates IOP Elevation in Mice

**Figure 1:**
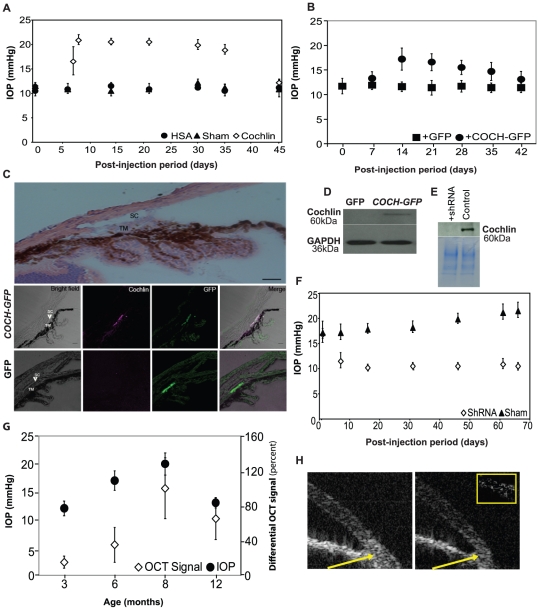
Elevated recombinant cochlin expression in mice TM is concomitant with the IOP elevation. (A) DBA/2J-Gpnmb^+^/SjJ mice (n = 42–48 for each vector at each time point, as indicated by the symbols) at six months of age were injected with a lentiviral vector bearing the COCH-GFP transgene or GFP alone (sham) or human serum albumin (HSA) in the anterior chamber (all under the control of a CMV promoter). IOP was recorded at the indicated time periods. (B) C57BL/6J mice at six months of age were injected with a lentiviral vector bearing the *COCH*-*GFP* transgene (n = 20) or *GFP* alone (n = 20) in the anterior chamber. The mice were followed and IOP recorded at the indicated time periods. (C) Representative immunohistochemical analysis of DBA/2J-Gpnmb^+^/SjJ mice eyes injected with *COCH-GFP* transgene or GFP alone. Top panel shows the anatomy of the anterior chamber stained with haematoxylin and eosin. SC = Schlemm's canal, TM = trabecular meshwork; Bar = 125 µm Bottom panels show eyes injected with COCH-GFP or GFP alone as indicated. The brightfield and antibody probing for cochlin (magenta), GFP (green) and merge image has been shown as indicated. Scale bar = 100 µm (D) Western analysis of the TM protein extract of the DBA/2J-Gpnmb^+^/SjJ mice injected with GFP alone or COCH transgene with IRES mediated GFP expression (COCH-GFP). GAPDH has been shown as a loading control. (E) Western analysis of the TM extracts of DBA/2J mice (8 months old) un-injected control or injected with cochlin shRNA. Coomassie blue stained gel shows total protein loading. (F) Downregulation of cochlin in DBA/2J cochlin^+/+^ mice decreases the mean IOP. DBA/2J cochlin^+/+^ mice (n = 38–45 for each vector at each point) at six months of age were injected with a lentiviral vector bearing cochlin shRNA or GFP alone in the anterior chamber. IOP was recorded at the indicated time periods. (G) Level of cochlin expression in the TM of DBA/2J cochlin^+/+^ mice correlates with the IOP. The level of cochlin expression was quantified at different ages (n = 10 at each age group) in vivo using the spectral domain OCT (experimental procedures) and IOP are represented by hollow and solid symbols respectively. (H) Representative SD OCT image of the anterior chamber angle a 6 month old DBA/2J cochlin^+/+^ mice before (left) and after (right) injection of infra-red (IR-800) dye coupled anti-cochlin antibody. The arrow indicates the region undergoing a change in intensity of signal before and after injection. The images obtained before and after injection were digitally subtracted for the region of interest (inset). Error bars (A, B, F and G) depict ± standard deviation.

Cochlin was overexpressed in DBA/2J-Gpnmb+/SjJ mice, which do not develop elevated IOP or glaucomatous neurodegeneration with age, to determine its role in IOP elevation. Mice injected with COCH transgene with IRES mediated GFP expressing (COCH-GFP) lentiviral vector into the anterior eye chamber showed a rise in IOP concomitant with cochlin expression, reaching a peak between 8–30 days. The IOP remains elevated upto 35 days post-injection in animals injected at the age of 6 months (Figure 1A). The IOP returned close to baseline after about 6 weeks although the cochlin expression remained almost the same (Figure S1A). The lowering of the IOP is consistent with lower aqueous production (data not shown) and is consistent with ciliary body hyalinization and shutdown in the end stages of glaucoma [11]. Control mice injected with GFP vector alone (sham) or human serum albumin (HSA) expression vector had no change in IOP compared to baseline (Figure 1A). Injection of exogenous cochlin (but not HSA; 10 µg) also results in IOP elevation in this strain (Figure S1B). IOP elevation with cochlin overexpression was also found in C57BL/6J mice (Figure 1B). Immunofluorescence studies confirmed cochlin expression in COCH-GFP injected eyes, whereas control GFP injected eyes demonstrated no cochlin expression (Figure 1C). Western blot analysis of TM extracts corroborated these findings (Figure 1D). Intracameral injection ensured that transfection covered a large swath of TM tract. The transfection was verified by detecting the GFP expression (Figure S1A) and robust GFP expression in vivo in real time (Figures S1C–S1F). Despite significant IOP elevation, the hematoxylin-eosin stained sections of COCH-GFP expressing eyes demonstrate open iridocorneal angles (Figure S1G).

To determine if downregulation of cochlin expression decreases IOP, cochlin shRNA or control GFP lentiviral vector was injected into the anterior chamber (Figure 1E) of DBA/2J mice eyes. Cochlin shRNA injection resulted in decreased cochlin expression in the angle (Figure 1E). DBA/2J mice normally demonstrate peak IOP elevation at 8 months of age [12]. Mice injected with cochlin shRNA at six months of age showed a decrease in IOP and maintained the decreased IOP level for the next two months compared to control injected eyes (Figure 1F). Cochlin levels in DBA/2J mice TM was assessed in vivo using spectral domain (SD) and recently developed [13] magneto-motive (MM; data not shown) optical coherence tomography (OCT) demonstrating a peak expression at 8 months coincident with peak IOPs (Figures 1G and 1H).

**Figure 2:**
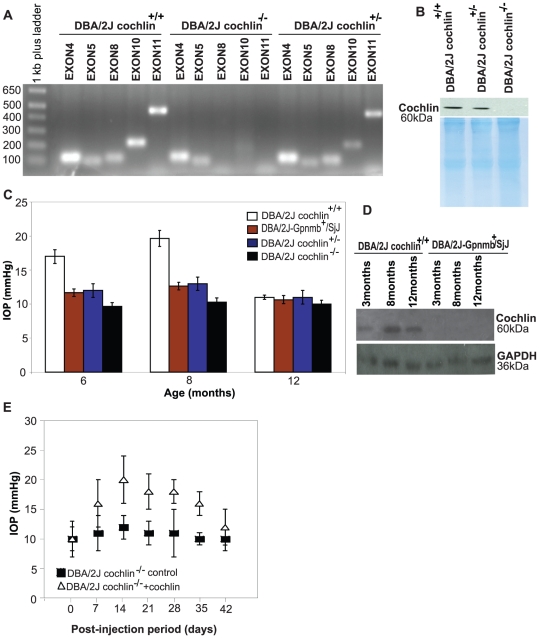
Down-regulation and/or disruption of cochlin expression in mice TM is commensurate with reduction in mean IOP. (A) Agarose gel (1.2%) showing the absence of PCR products for exons 8, 10 and 11 in the DBA/2J cochlin^−/−^ mice. These exons are present in DBA/2J cochlin^+/+^ and cochlin^+/−^ mice. DNA was isolated from mice tail and subjected to PCR amplification for the indicated exons. (B) Western blot analysis of TM extract of DBA/2J cochlin^+/+^, DBA/2J cochlin^+/−^ and DBA/2J cochlin^−/−^ mice showing the complete absence of cochlin in DBA/2J cochlin^−/−^ mice. Coomassie blue stained gel shows equal loading of protein in all the lanes. (C) Intraocular pressure (IOP in mmHg) in different mice strains at indicated ages (n = 35–45 for each strain at each age group). The DBA/2J cochlin^+/+^ mice show a higher mean IOP as compared to DBA/2J-Gpnmb^+^/SjJ, DBA/2J cochlin^+/−^ and DBA/2J cochlin−/− mice at 6 months and at 8 months of age. The difference is most marked at 8 months of age. By 12 months, the mean IOP is similar for the different strains. (D) Western blot analysis of the TM protein extract from the DBA/2J cochlin^+/+^ and DBA/2J-Gpnmb^+^/SjJ at the indicated ages, probed for cochlin. GAPDH has been shown as a loading control. (E) Generation 10 (Gen 10) DBA/2J cochlin^−/−^ at six months of age were injected with a lentiviral vector bearing the COCH-GFP transgene (n = 20) or GFP alone (n = 20) in the anterior chamber. The mice were followed and IOP recorded at the indicated time periods. Error bars (C and E) depict ± standard deviation.

The role of cochlin in IOP elevation and its peak at 8 months was studied by abrogating cochlin. C57BL/6J mice with targeted disruption (knockout) of COCH [14] were bred with DBA/2J mice to derive cochlin−/− on a DBA/2J background [see methods for details]. DBA/2J cochlin−/− mice lacked the expression of cochlin exons 8, 10 and 11 (Figure 2A). TM protein extracts (the TM tissue was dissected under microscope, however, it is likely to retain some contamination from sclera and cornea) from DBA/2J cochlin−/− mice lacked cochlin expression in contrast to its presence in DBA/2J cochlin+/+ and DBA/2J cochlin+/− mice (Figure 2B).

Mean IOP rose from 17 to 19.7 mm Hg as DBA/2J cochlin+/+ mice age from 6 to 8 months and then decreased to 12 mm Hg by 12 months of age (Figure 2C), consistent with previous reports [15]. DBA/2J cochlin−/− mice had a mean IOP of 10–11 mm Hg and lacked IOP elevation exhibited by DBA/2J cochlin+/+ mice at 8 months of age. Cochlin expression increases in the DBA/2J cochlin+/+ mice with age until approximately 8 months and decreases slightly thereafter (Figure 2D), DBA/2J-Gpnmb+/SjJ control mice lacked cochlin expression in their TM. Overall these results suggests concomitant IOP elevation and conversely maintenance of IOP within normal range in DBA/2J-Gpnmb+/SjJ and DBA/2J mice is due to cochlin overexpression and down-regulation, respectively.

Re-introduction of COCH gene expression in DBA/2J cochlin−/− mice leads to increased IOP, in contrast to control GFP injected mice (Figure 2E). Increased expression of cochlin was commensurate with optic nerve damage as assessed by paraphenylenediamine (PPD) staining and its down-regulation prevented IOP associated neurodegeneration (Figures S2A–S2D). Seventy five percent of the DBA/2J mice developed severe optic nerve damage at one year. The corresponding number (mice with severe optic nerve damage) for the DBA/2J cochlin+/− DBA/2J cochlin−/− and DBA/2J-Gpnmb+/SjJ was 5%, 1% and 3% respectively (Figure S2E). Taken together these results demonstrate that cochlin plays a key role in IOP elevation in DBA/2J mice, consistent with its overexpression in human glaucomatous but not control TM [8]. Cochlin overexpression correlates with elevated anterior segment pressure (ASP) in monkey and porcine cultured anterior segments [16], [17] and its downregulation results in decreased IOP in normotensive rabbits [18].

### Cochlin is a potental mechanosensing molecule

The fluid flow changes must be sensed by cells in order to regulate the structure of the TM that allows passage of aqueous humor and regulate its flow. Aqueous humor is a Newtonian fluid [19] where the shear force is proportional to multiplicative of cross section area and rate of fluid flow and inversely proportional to path length of flow with viscosity acting as proportionality constant. The difference in the viscosity of aqueous humor and other factors except flow rate between glaucomatous and non-glaucomatous eyes has been reported to be insignificant [20]. The fluid shear or the force experienced by the TM cells is primarily proportional to the rate of aqueous outflow. The force required for the keeping the aqueous in flow varies linearly with the flow rate. The flow rate shows diurnal variation, thus TM cells experience different fluid shear at different times of the day [21]. Although the aqueous flow rates for normal and glaucomatous eyes are the same when the daytime flows are compared, the nighttime flow is significantly higher in glaucomatous eyes [22]. Though this increased flow may not have profound effects on TM cells or ECM over few days, chronic exposure over the years may result in significant cumulative changes.

**Figure 3:**
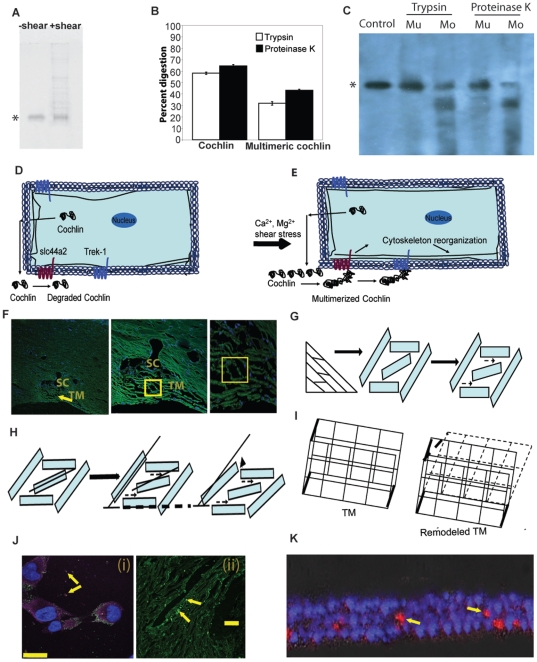
Cochlin mechanosensing and proposed model of potential associated TM changes. (A) Western analysis of purified recombinant cochlin (*) using anti-cochlin antibody before after sheer stress, revealing shear stress-induced mulitmers. Cochlin was subjected to fluid shear of 3 µl/min for 150 cycles. (B) Comparison of proteolytic digestion (in percentage) of native (left side) and multimerized (right side) cochlin by trypsin (open bars) and proteinase K (filled bars) showing slower digestion of multimerized cochlin. Digestion of multimerized and native cochlin (10 µg) was performed using 0.01 µg/10 µg Trypsin or 0.05 µg/10 µg Proteinase K. After incubation at room temperature, the samples were boiled using 1 mM DTT at 100°C for 1 minute and analyzed on SDS-PAGE. Densitometric analyses data from three independent experiments (mean± standard deviation) has been presented. (C) A representative Western analysis of native and multimerized cochlin subjected to subcatalytic amounts of trypsin and proteinase K digestion for 10 minutes at room temperature. Purified recombinant cochlin (*), native monomeric cochlin and multimeric cochlin (initial amount 1 µg) has been depicted as Mo and Mu respectively. Digestion was performed by 0. 1 µg/10 µg Trypsin or 0.5 µg/10 µg Proteinase K. Digestion was stopped by heating at 100°C, separated on a 10% reducing SDS-PAGE and probed with chicken polyclonal antibody against cochlin. Proposed model of cochlin mechanosensing and associated global change in TM. (D) Illustration depicting that the cochlin constitutively secreted by the normal TM cells is degraded by the proteases (dashed lines denoting degraded proteins). (E) Cochlin in the presence of fluctuating shear stress (or elevated divalent cations), forms multimers which are resistant to proteolysis. Multimerized cochlin may potentially interact with the transmembrane proteins TREK-1 or slc44a2 either directly, functionally, or indirectly leading to events that may result in cytoskeletal reorganization. (F) Confocal microscopy image showing the sieve like structure of TM (arrow). A representative area of TM with rectangular arrangement of cells is highlighted by a yellow box. SC = Schlemm's canal; TM = trabecular meshwork. The magnified view of rectangular arrangement of cells highlighted by a yellow box also has been shown. (G) The line diagram depicts the filter like structure of the TM. A magnified view mimicking one such rectangle as in (F) with an additional cell in the middle of the rectangle has been shown. Notice the change in orientation of the middle cell opens up interstitial space for additional fluid flow indicated by dashed arrows. (H) When incremental space opening is insufficient, larger orientation changes by cells in the middle and those forming rectangle are necessary to increase fluid flow. Newly opened space is indicated by arrow head. The dark lines and dark dashed lines are to indicate orientation changes. (I) Cartoon illustrating concerted orientation changes in several individual cells (dashed lines) leading to a global change in the structure of the sieve like TM. (J) Confocal microscopy image of cochlin transfected TM cells (left; i) expressing cochlin (pink) and tiny aggregates of cochlin (arrows) secreted into the media. These small cochlin aggregates coalesce (arrow) to form larger deposits as shown in human glaucomatous TM probed for cochlin (green; right; ii). Scale bar = 25 µm (left) and 50 µm (right). (K) Confocal microscopy image of trilayer of cochlin transfected TM cells on a PVDF membrane exposed to continuous fluctuation in fluid shear stress in an Ussing type chamber showing the development of cochlin deposits (red; arrows).

Cochlin, at the protein level, possesses a short signal peptide and two von Willebrand factor A-like (vWFA1 and vWFA2) domains. The vWFA domain present in ECM proteins is associated with fluid shear responsiveness [23]. We previously showed that purified recombinant cochlin is shear responsive [24]. Fluid shear induces cochlin multimerization (Figure 3A). In an effort to model the in vivo fluid flow, we used a microsyringe and ultra micro pump subjecting purified exogenous cochlin to different rates of fluid flow. Purified cochlin (10 µg) was subjected to flow rates of 2.5 µl/min, 3.0 µl/min or 5.0 µl/min for 50, 100 or 200 cycles of injection and aspiration (see methods for details). At 2.5 µl/min the cochlin shows minimal multimerization after 50 or 100 cycles but shows 24±1.5%multimerization after 200 cycles. In contrast, at 3.0 µl/min the cochlin shows 5±1.35%, 15±2.5%, 78±6.8% multimerization after 50 100 and 200 cycles respectively. At 5.0 µl/min observed cochlin multimerization was 47±2%, 81±3.8%, and 93±4.9% after 50, 100 and 200 cycles respectively (Figure S3A, S3B). Multimerized cochlin undergoes slower proteolysis compared to monomeric cochlin (Figure 3B–C). Cochlin is secreted in the normal TM as well but likely to be quickly degraded by the proteases. We provide evidence that in increased fluid shear leads to cochlin multimerization (Figure 3A; S3). This multimerized cochlin is resistant to proteolysis (Figure 3B, C) and potentially accumulates in the TM ECM in glaucomatous eyes. The multimerization of cochlin is consistent with molecular mechanosensing observed for other proteins such as vWF [25].

## Discussion

TM cells function in an environment of continuous varying mechanical and fluid shear forces [26]. Our understanding of molecular mechanosensors and their role in transduction of stimuli into cellular biochemical signals that ultimately regulate cellular function [25] is very limited. Mechanical forces are possibly transmitted through protein-protein interactions linking the ECM via transmembrane proteins to cytoskeletal proteins [27], [28].

Fluid shear, cyclic strain and osmotic shock are forms of biomechanical stress [29] which cause changes in TM gene expression [6], [30]. Reversible multimerization of vWF [23], [25] and cochlin caused by shear stress (Figure 3A; Figure S3) can act as potential mechanosensing mechanisms [25]. We hypothesize that normal constitutively secreted cochlin in ECM is eventually degraded (Figure 3C, D). We found fluid shear fluctuations, cations and/or osmolarity to result in the formation of proteolysis-resistant multimeric forms of cochlin (Figures 3A–C; Figure S3). We propose that shear-induced multimeric cochlin resides for a longer period in the TM (Figure 3D, E).

We hypothesize that the fluid shear responsive property of cochlin plays a role in tissue remodeling, in consonance with transmembrane shear transducing proteins (SACs). Expression of cochlin has been shown to result in co-expression of an SAC, TREK-1 in the primary TM cells [10]. In the inner ear protein extract, cochlin has been shown to co-precipitate with solute transporter slc44a2 [31]. A direct or functional interaction with either TREK-1 or slc44a2 is yet to be shown in the TM cells. We propose that mechanosensing and mechanotransduction may induce alterations in cell shape and motility within the TM. Cultured primary human TM cells demonstrated syn-expression of cochlin and TREK-1 in the filopodia at the very initial stages at the leading edges of the protrusions [10]. Voltage sensitive dye, bis-(1, 3-dibutylbarbituric acid) trimethine oxonol (DiBAC) intake studies [32] further showed that TREK-1 interaction with cochlin is functional resulting in activation of TREK-1 channel and concomitant changes in TM cell shapes [10]. TREK-1 mediated morphological changes in endothelial cells were shown to be independent of its channel activity [33], which is similar for TM cells as well [10]. Cellular level syn-expression and functional interaction of cochlin and TREK-1 is consistent with their role in mechanosensing and mechanotransduction. Mechanosensing induced changes in cell shape and motility should create open spaces for fluid outflow. Potential modulation of cell shape and motility and overall space expansion mediated by cochlin should induce overall TM filter changes and result in increased fluid flow.

SACs (TREK-1) are activated by shear and osmolarity [34] and they contribute to pressure regulation in the kidney and the cardiovascular system [35]. Although TREK-1 as well as slc44a2 mRNA are present in TM cells (Unigene), however, they have not been implicated in aqueous outflow or pressure control in the TM. Our findings implicate ECM mechanosensing by cochlin with potential transmembrane mechanotransduction. Our results suggest mechanotransduction by cochlin possibly involving TREK-1 [10] of fluid flowing through the ECM. Such mechanotransduction can potentially modulate the expression of several cytosolic proteins, for example, profilin I, which in turn may cause cytoskeletal remodeling [36] concomitant with increased outflow facility [37] and fluid flow resulting in reduction of IOP [38], [39].

We propose a model where such mechanosensing and mechanotransduction enables some TM cells (Figure 3F) to undergo minor orientation changes in the TM aqueous filter (Figure 3G). Within the filter-like TM arrangement (Figure 3F), some cells secrete cochlin and enable a cell to undergo incremental change in orientation which increase fluid outflow (Figure 3G; space opening indicated by dashed arrows). We envisage that when these incremental orientation changes are not sufficient, coordinated movements of cells in oblique and horizontal planes occur and open additional fluid escape routes (Figure 3H; arrows and arrowhead indicate newly opened space). We predict that concerted changes in many cells simultaneously will result in overall change across the TM (Figure 3I). Observed cochlin and TREK-1 co-expression [10] are consistent with role of mechanosensing and mechanotransduction molecules and initiation of these changes (Figures 3D–3I). Once initiated, further and progressive cellular changes result in TM remodeling and likely enhanced fluid flow across cell layers. Maximum resistance to aqueous outflow is localized to the juxtacanalicular tissue of the TM [40]. Studies suggest TM cells dynamically regulate outflow facility by rearranging their cytoskeleton, thus enhancing aqueous outflow [41].

We also found that prolonged overexpression of cochlin results in formation of cochlin deposits in cell culture (Figure 3J, i) that resembles cochlin deposits observed in vivo (Figure 3J,ii). We also found that frequent fluctuating fluid shear result in similar formation of cochlin deposits in vitro (Figure 3K) that resembles in vivo cochlin deposits (Figure 3J,ii). We used an Ussing type chamber attached to a peristaltic pump was used to apply shear stress equivalent to that experienced by TM cells when subjected to a flow rate of 3.0 µl/min (Figure 3K). Presence of deposits have been reported in collector channels in glaucomatous eyes [42]. Such collector channel blockage augmented by contributions from various factors, such as fluctuating fluid shear and oxidative stress, lead to an irreversible progressive pathological state in the eye such as glaucoma [43], a family of late onset irreversible blinding optic neuropathies affecting over 60.5 million people worldwide [44]. Elevated IOP and diurnal fluctuation in IOP [5] are major risk factors in glaucoma. TM cells in glaucomatous eyes experience a significant level of fluid shear change due to IOP fluctuation. IOP reduction acts as a neuro-protectant delaying glaucoma progression [45]. The development of drugs which increase aqueous drainage via the TM pathway is needed. The present work shows involvement of cochlin in regulation of intraocular pressure likely by modulating TM outflow. In the future further detailed mechanistic insight along these lines may lead to development of more efficacious intervention strategies.

## Materials and Methods

### Ethics Statement

The work was conducted adhering to the guidelines of the Institute Review board of the University of Miami. All human samples were handled in keeping with the principles expressed in the Declaration of Helsinki. All experiments with the human samples were conducted at the SKB lab and the protocol was approved by the Institute Review board of the University of Miami. A written informed consent was obtained from all patients undergoing trabeculectomy for POAG and donating the tissue so obtained for research. Cadaveric human eyes were obtained from Bascom Palmer Eye Bank with the approval of Institute Review board of the University of Miami. Human TM cell culture protocol was approved by the Institute Review board of the University of Miami.

### Tissue Procurement and Mice Housing

Adult mice from inbred strains DBA/2J and DBA/2J-Gpnmb^+^/SjJ were obtained from the Jackson Laboratory (Bar Harbor, ME). Mice with targeted cochlin knockout allele [14] were bred onto the DBA/2J background to produce DBA/2J cochlin^+/−^ and DBA/2J cochlin^−/−^ mice. We established colonies of DBA/2J, DBA/2J cochlin^+/−^, DBA/2J cochlin^−/−^ and DBA/2J-Gpnmb^+^/SjJ mice at the Animal Facility of the University of Miami, Miller School of Medicine (Miami, FL), in a controlled environment under a Institutional Animal Care and Use Committee (IACUC) approved protocol. The animals were maintained in rooms with 12 h light/dark cycles and fed ad libitum. TM tissue samples were carefully removed surgically under a dissecting microscope by a trained ophthalmologist following euthanasia with carbon dioxide, and adhering to animal procedures approved by the IACUC (protocol numbers 06-027; 09-008; IBC 06-007; 09-001).

Human eyes from normal and POAG donors, all between 40 and 85 years of age (Table S1), were used in this study, and were obtained through the Bascom Palmer Eye Bank (BPEI) and National Disease Research Interchange (NDRI). The protocol for use of human tissue was approved by the Institutional Review Board of the University of Miami. Eyes were enucleated within 12 h of death and stored at −80°C until TM tissue was isolated by dissection. Normal control eyes were from donors with no visual field defects, no evidence of glaucoma, and without central nervous system abnormalities. Fixed human TM tissues used for immunohistochemistry were obtained from the Eye Donor Program of the Foundation Fighting Blindness (Owings Mills, MD). Glaucomatous eyes and tissues were from clinically documented POAG donors. Glaucomatous TM tissues (∼1–2 mm^3^) were obtained by trabeculectomy from POAG patients in the BPEI and Mundorf Eye Center (Charlotte, NC) with institutional review board approval. Human tissue obtained by trabeculectomy consisted predominantly of TM; however, possible contamination with small amounts of surrounding tissue (e.g. sclera) cannot be excluded. TM cells for cell culture were isolated from the rim tissue associated with corneas used for transplantation at the BPEI and were obtained from healthy human eyes within 3 h of death and stored until use in Optisol-GS medium (Chiron Vision, Claremont, CA).

### Intraocular Pressure Measurement

The mice were anaesthetized using intraperitoneal injection (0.1 µl) of ketamine (100 mg/kg) and xylazine (9 mg/kg). One mouse was anaesthetized at a time and IOP was measured as soon as the mouse failed to respond to touch. All care was taken to ensure that the mice were in a similar level of anesthesia when the IOP measurements were made. To measure the IOP, hand held tonometer, TonoLab (Colonial Medical Supply, Franconia, NH) was used. To ensure accuracy of the measurements, TonoLab measurements were confirmed using anterior chamber cannulation as described previously [12] in a subset of mice. The two IOP measurement methodologies correlated very closely in most cases.

### Quantification of Cochlin Expression in Mice TM Using Western Blot Analysis

Mice were euthanized at the indicated ages and the eyes were immediately enucleated. TM was dissected out of the enucleated eyes under a dissecting microscope by a trained ophthalmologist. Such dissected tissue can have contamination from surrounding tissue. Previously with immunohistochemistry we have shown the presence of cochlin largely to remain confined to TM [24]. Each mouse TM sample was used for preparation of protein extract individually and analyzed individually. Thus obtained TM was finely minced and proteins were extracted using 50 mM Tris-HCl, pH 7.5, 125 mM NaCl and 0.1% genapol (cat# 345794, EMD Biosciences, La Jolla, CA).The protein extract was subjected to Western blot analysis. For Western blot the proteins were separated on 4–20% Tris-glycine gel (cat# EC6028BOX, Invitrogen, Carlsbad, CA) and then transferred to polyvinylidene fluoride membranes (PVDF) (cat# 162-0219, BioRad Labratories, Hercules, CA). For cochlin identification, custom chicken polyclonal antibodies against cochlin peptides (KR LKK TPE KKT GNK DC from cochlin coding region 147–162 designated as hCochlin# 1; ZCZ TYD QRT EFS FTD YST KEN; from cochlin coding region 412–429 designated as hCochin#2; and CZ DDL KDM ASK PKE SH from cochlin coding region 358–371 designated as hCochlin#3, Aves labs Inc., Tigard, OR) were used [3]. A secondary antibody conjugated to horseradish peroxidase (goat anti-chicken cat# H-1004, Aves Labs Inc.) was added and proteins were detected using enhanced chemiluminescent substrate (cat# 32106, Pierce Thermo Fisher Scientific Inc, Rockford, IL). GAPDH (anti-GAPDH cat# ab22556, Abcam, Cambridge, MA) was used as a loading control.

### Optic Nerve Analysis

Mice were euthanized at 12 months of age and the eyes were immediately enucleated and fixed in 2% Glutraldehyde and left overnight at 4°C. To asses for glaucomatous damage, the optic nerves were sectioned and subsequently stained with paraphenylenediamine (PPD) Images were taken using Zeiss Axiostar Plus CYL#621 microscope (Carl Zeiss Microimaging Inc, Thornwood, NY).

To quantify the percentage of mice suffering from mild, moderate or severe optic nerve damage, PPD stained sections of each mouse were graded by three observers in a blinded fashion independent of each other. Three sections from each mouse (n = 25 in each group) were graded and given a score of 1–10 with one being the least severe and ten being the most severe damage. The scores from the two observers were averaged. Mice getting a final score of 0–4 were labeled as having mild damage, 5–7 moderate damage and 8–10 severe damage.

### Mice Intraocular Injections

Mice were anaesthetized as for IOP measurement. A topical anesthetic (tetracaine hydrochloride 0.5%, Bausch & Lomb) was applied to the desired eye. 1 µl of the lentiviral vector bearing the gene of interest (*COCH-GFP* transgene, *GFP* alone or cochlin shRNA) was injected using a 36G beveled needle (cat# NF36BV-2, World precision instrument, Sarasota, FL) mounted on 10 µl microsyringe (cat# NanoFil 300329, World precision instrument). An UltraMicroPump II (World precision instrument's UMP2 and UMC4) was used to precisely control the volume of injection. The needle was entered at the limbus and care was taken not to traumatize the iris or the lens. Mice with injury to iris or lens were excluded from the study. As the needle was withdrawn after injection, a cotton tip applicator was applied for about 1 minute to prevent the virus/aqueous reflux.

### Mice Genotyping

C57BL/6J mice with targeted disruption (knockout) of *COCH* [14] were bred with DBA/2J mice to derive cochlin^+/−^ on a DBA/2J background. C57BL/6J cochlin knockout mice were generated by eliminating exons (7–11) corresponding to amino acids 147–552 encompassing cochlin vWA domains [14]. Heterozygotes (cochlin^+/−^) were subsequently bred to derive cochlin^−/−^ (cochlin KO) in a DBA/2J background. The genotyped disrupted cochlin allele was bred into a DBA/2J background for greater than 10 generations to generate cochlin deletion in a DBA/2J background. The 10^th^ generation cochlin^−/−^ so obtained were labeled as Gen 10. DNA isolated from the mice tail was subjected to PCR amplification for exons 4, 5, 8, 10 and 11 at each generation.

Mice were anaesthetized as described above for IOP measurement. Genotyping was performed on mice approximately 3 weeks of age. Mice tails were tattooed (AIMS, ATS-3 general rodent tattoo system) for identification. Then 2.5–3.0 mm of tail was clipped. The tail was cauterized using Kwik-Stop styptic powder for hemostasis. Three hundred µl of Lysis reagent (Direct PCR-Tail, cat#102-T, Viagen Biotech, Los Angeles, CA) and 9 µl of 10 mg/ml proteinase-K solution (cat# P6556, Sigma-Aldrich, St. Louis, MO) was added to the excised tail and incubated overnight at 55°C. The following morning the tail samples were heated for 45 minutes at 85°C. DNA isolated from the tail was then subjected to polymerase chain reaction (PCR) amplification for exons 4, 5, 8, 10 and 11. PCR products were run on a 1.2% agarose gel (Ultra-pure agarose, cat# 16500-100, Invitrogen) to assess for the presence of the above exons. Following primer pairs were used for identifying DBA/2J cochlin^−/−^ mice:

Exon 4: Sense: 5′-CCATTCCTGTCACCTGCTTT; Antisense: 5′-ATGCTGGACACTGACGCATA.

Exon 5: Sense: 5′-GGAGTGATTGGCACCTCAG; Antisense: 5′-TCGGGAAAGCATCTGAGACT.

Exon 8: Sense: 5′-GGCAGACATTGCATTTCTCA; Antisense: 5′-ACGTGTGGTCCTTCTGTTCC.

Exon 10: Sense: 5′-AAAGCCTTGAAGCACACTGC; Antisense: 5′-CTTGTCAACAAATGCAACATCT.

Exon 11: Sense: 5′-ATCACATGCCCAACTGGTTT; Antisense: 5′-GCCTCGGACATCATCATAGG.

The sizes of different exons are as follows Exon 4: 157 bp, Exon 5: 134 bp, Exon 8: 148 bp, Exon 10: 227 bp and Exon 11: 517 bp.

The cochlin KO mice were bred onto DBA/2J background for 10 generations (Gen10) as described below:

Step 1. Crossed DBA/2J Coch^+/+^ X Coch KO^†^ to obtain Gen1 (Heterozygote Coch^+/−^)

Step 2. Crossed Gen1 X Gen1 to get Gen1*

Step 3. When Gen1* is genotyped and its KO status was established, it was termed as Gen1 KO.

Step 4. Crossed Gen1KO X DBA/2J Coch^+/+^ which was termed Gen2

Step 5. Crossed Gen 2 X Gen 2 to get Gen2*

Step 6. When Gen2* is genotyped and its KO status was established, it was termed as Gen2 KO.

Step 7. Crossed Gen2KO X DBA/2J Coch^+/+^ which was termed Gen3

Step 8. Repeated steps 4 to 7 till Gen 10 KO has been obtained.

(† Coch KO was obtained as a research gift from Dr. C. Stewart at National Institutes of Health [14]).

### Quantification of Shear Stress And Cochlin Multimerization

Cochlin was purified according to the established protocols [3]. 10 µg of the purified exogenous cochlin was taken and subjected to fluid shear using a 10 µl microsyringe (cat# NanoFil 300329, World precision instrument) and UltraMicroPump II (World precision instrument's UMP2 and UMC4). The flow was adjusted to 2.5 µl/min or 3.0 µl/min or 5.0 µl/min was maintained for 50 or 100 or 200 cycles of injection and aspiration at each of the flow rates. The injection and aspiration flow rates were identical, all operations were performed at a constant temperature of 25±0.5°C (room temperature was controlled at this level during these experiments) the samples were emptied into an eppendorf tube. The protein samples so obtained were subjected to Western blot analysis and the extent of multimerization was quantified by densitometric analysis. Protease digestion of multimerized and native cochlin (10 µg or 1 µg) was performed using either 0.1 µg/10 µg or 0.01 µg/10 µg Trypsin (sequencing grade porcine trypsin; catalog#V5111 Promega Corporation, WI) or 0.5 µg/10 µg or 0.05 µg/10 µg Proteinase (catalog#25530-015, Invitrogen, Carlsbad, CA) in buffers recommended by the manufacturer. After incubation at room temperature, the samples were boiled using 1 mM DTT at 100°C for 1 minute and analyzed on SDS-PAGE.

Shear force (F) = η Av/l, for aqueous humor, where η is the viscosity of the fluid, A is the cross sectional area of the chamber through which the fluid is flowing and l is the length of the chamber. The factor l for aqueous humor is the distance between the ciliary epithelium and the episcleral veins. For aqueous humor outflow whether in normal or in glaucomatous eyes, η, A and l remain constant. The flow rate (v) is the greatest determinant of fluid shear or Force.

### Immunohistochemistry

DBA/2J-Gpnmb^+^/SjJ mice were euthanized and the eyes were enucleated immediately. The globes were fixed using 4% paraformaldehyde (cat# 19943, USB Corp, Cleveland, OH) overnight, washed with 1XPBS 3 times for 10 m per wash, placed in 15% sucrose for 4 h and then switched to 30% sucrose for another 4 h. The globes were then placed in a cryomold (cat# Tissue-Tek 4565, Sakura Finetek, USA) and embedded in O.C.T. (Optimum Cutting Temperature) compound (cat# Tissue-Tek 4583, Sakura Finetek, USA) using liquid nitrogen. The embedded globes were then sectioned and subjected to immunohistochemcal analysis probing for cochlin (hCochlin#3, Aves Labs Inc.) and GFP (cat#, 600-301-215 Rockland Immunochemicals Inc., Gilbertsville, PA).

### Cochlin Transgene and Cochlin shRNA Lentivirus Production

To overexpress cochlin in the TM of congenic DBA/2J mice, cochlin transgene lentivirus was constructed in the HEK-293T [cat# 293T/17 (CRL-11268), ATCC, Manassas, VA] cells. Cochlin expression clone (cat# EX-Q0226-Lv31, GeneCopoeia Inc., Rockville, MD) was packaged into a lentiviral vector by using the Lenti-Pac FIV expression packaging kit and the protocol provided by the manufacturer. This protocol typically yielded 10^7^ ifu/ml of the recombinant lentivirus. The *COCH* gene used to make the *COCH* expression vector was human (NM_004086).

To down-regulate cochlin expression in the TM of DBA/2J mice cochlin shRNA was constructed in HEK-293T cells using the Trans-Lentiviral™ GIPZ Packaging System (cat# TLP4614, Open Biosystem, Huntsville, AL) and the protocol provided by the manufacturer. This typically yielded a viral stock of 10^8^ tranduction unit (TU)/ml. For control experiments a lentiviral vector expressing GFP was constructed in a similar fashion. The shRNA used was set of 5 clones. The sequence of the clones used is shown below.

TGCTGTTGACAGTGAGCGCGGGCAGCGCCGATTTAATTTATAGTGAAGCCACAGATGTATAAATTAAATCGGCGCTGCCCATGCCTACTGCCTCGGA (catalog no. V2LHS_172911);

TGCTGTTGACAGTGAGCGCGCTTCTTTCACAGTAACTAAATAGTGAAGCCACAGATGTATTTAGTTACTGTGAAAGAAGCATGCCTACTGCCTCGGA (catalog no. V2LHS_172909);

TGCTGTTGACAGTGAGCGCCAGGTAAACGACTAAAGAAAATAGTGAAGCCACAGATGTATTTTCTTTAGTCGTTTACCTGTTGCCTACTGCCTCGGA (catalog no. V3LHS_369645);

TGCTGTTGACAGTGAGCGAACGGTAGATGCTGGAGTAAGATAGTGAAGCCACAGATGTATCTTACTCCAGCATCTACCGTGTGCCTACTGCCTCGGA (catalog no. V3LHS_369649);

TGCTGTTGACAGTGAGCGATCGGACATTGGTGCCAAGATATAGTGAAGCCACAGATGTATATCTTGGCACCAATGTCCGAGTGCCTACTGCCTCGGA (catalog no. V3LHS_369647).

### TM Cell Culture Experiments

Primary human TM cells were cultured from cadaveric corneo-scleral sections obtained from the Bascom Palmer Eye Bank (BPEI) and Mundorf Eye Centre (Charlotte, NC). The cells were isolated through a blunt dissection of the area containing and adjacent to the canal of Schlemm, followed by 2 h digestion in 1X PBS (cat# 21-030-CV, Mediatech Inc.) suspension of 20% 0.01 µg/µl collagenase-A (cat# LS004194, Worthington, Lakewood NJ). The blunt dissection and the proteolytic treatment were performed inside a 12 well culture plate (cat# 665-180 Greiner Bio-One, Neuburg, Germany). Culture media containing [DMEM 1X (cat# 10-017-CM, Mediatech Inc.), 10% heat inactivated fetal bovine serum (FBS) (cat# 35-016-CV, Mediatech Inc.), 0.5% 1.7 mM L-Glutamine (cat# G6392, Sigma-Aldrich), 1% Antibiotic-Antimycotic solution (cat# 30-004-CL, Mediatech Inc.)] was added after 2 h to terminate digestion. A sterile microscopy slide (cat# 56700-194, VMR) was placed on top of the tissue fragment to ensure bottom-contact and immobility inside the media well. The sections were cultured at 37°C, 5% CO_2_ cell culture incubator. Culture was washed with 1X PBS 7 days later to remove tissue debris; media change occurred every 3–4 days. Thus obtained cells were trypsin treated (cat# 25-050-Cl, Mediatech Inc.) and accordingly distributed the day before the transfection. Transfection complexes were created using a ratio of 0.4 µg/µl of respective vector DNA to the transfection agent (Lipofectamine 2000, cat# 11668-019, Invitrogen), prepared according to the manufacturer's instructions. Following the addition of the complexes to the selected culture wells, the transfection reactions were allowed to take place over a 2.5 h span, after which they were terminated through the addition of cell culture media.

Trabecular meshwork cells were cultured as described above on the PVDF membrane. Before plating the cells, a layer of collagen matrix (∼0.1 mg per layer; Rat Tail Collagen, cat# 354249, BD Biosciences, San Jose, CA) was formed on the membrane to facilitate cell adherence. The cells were allowed to form a confluent monolayer over a period of 16–24 h, following the addition of another layer. This process was repeated to ultimately achieve a confluent tri-layer of cells. Primary TM cells of the each layer for all multilayer cell containing experiments were transfected as described previously [10].

To demonstrate the formation of cochlin deposits in vitro, cochlin transfected TM cells were cultured on a PVDF membrane (Pall Life Sciences) with a pore size of 0.45 µM in a series of three confluent layers as described above. The membrane was then placed between the two hemi-vessels of the Ussing-type chamber connected to a standing column of serum-free cell culture media (DMEM 1X, Mediatech Inc) which was allowed to pass through the membrane by gravity, producing shear stress upon the cell layers as a result of the passage. Thus arranged setup was maintained for 12 h, following which the membrane was embedded in optimum cutting temperature medium and solidified using liquid nitrogen to permit sectioning. Obtained sections were subjected to immunohistochemical analysis probing for cochlin protein using cochlin primary antibody (usually hCochlin#3, Aves Labs Inc.), followed by respective secondary antibody incubation (A647: A21449, Invitrogen) and imaged using the confocal setup (Leica TSP5; DM6000 B).

### Spectral Domain Optic Coherence Tomography (SDOCT)

Mice were anaesthetized as for IOP measurement and a drop of topical anesthetic (tetracaine hydrochloride 0.5%, Bausch & Lomb, Tampa, FL) was applied to the desired eye. Custom generated 2 µl anti-cochlin antibody (hcochlin#3, Aves Labs Inc.), coupled to an infra-red dye with an absorption peak at 800 nm (cat# CUST154, Rockland Inc.), was injected into the anterior chamber of mice eyes. The anesthetized animals were restrained on a mounting tube, which was fixed on a 6-axis movable platform for imaging. An ultra-high resolution (∼3 µm) SDOCT was used to scan the mouse. The mice were imaged before injection and 30 minutes post-injection using the SDOCT. This analysis was performed on 3, 6, 8 and 12 month old DBA/2J mice. The images obtained before and after injection were subtracted digitally and the resultant images were analyzed for quantifying the OCT signal intensity using NIH ImageJ (v.1.43u) software which is representative of the amount of cochlin present. We imaged 10 mice each at 3, 6, 8 and 12 months of age. The maximum signal intensity (found in mice at 8 months) was regarded as 100% and all the readings are expressed relative to this intensity.

### In Vivo Imaging of GFP in TM

DBA/2J-Gpnmb^+^/SjJ mice injected with the lentiviral vector bearing the *COCH-GFP* transgene (GeneCopoeia Inc.) were imaged to document the *GFP* expression in-vivo. Images were taken before injection as well as, 1 and 2 weeks post-injection. Images were taken with the Micron III Microscope (Phoenix Research Laboratories, Inc, San Ramon, CA) with a trabecular meshwork (TM) attachment. The TM attachment consists of a 5-Watt white light LED source, filter wheel, and gonio-prism specifically designed for mice (Ocular Instruments, Inc., Bellevue, WA). Hypromellose solution (cat# NDC 17478-064-12, Akorn, Lake Forest, IL) was used as a coupling fluid between the camera lens and the cornea. Images obtained were subjected to uniform background subtraction.

## Supporting Information

Figure S1 ***COCH-GFP*** **transgene injection leads to cochlin expression in-vivo.** (A) Western analysis of the TM protein extract (10 µg) of the DBA/2J-Gpnmb+/SjJ mice injected with *COCH* transgene on days 7, 21 and 42 post-injection. GAPDH has been shown as a loading control. The lower panels indicate Western blot from control animals as indicated. (B) DBA/2J-Gpnmb+/SjJ mice (n = 45) at six months were injected (1 µl) with 10 µg of purified human serum albumin (HSA), Cochlin or PBS (sham) in the anterior chamber as indicated every 72 hours up to 44 days. (C) Image of a living mouse anterior chamber angle showing the cornea, iris and the TM (arrow). (D–F) In vivo imaging of GFP expression in mice TM. Before *COCH-GFP* injection [DAY 0, (D)] no fluorescence is seen in the TM but at DAY 7 (E) and DAY 14 (F) post-injection, GFP expression (arrow) can be visualized. (G) Hematoxylin-eosin stained images of DBA/2J-Gpnmb^+^/SjJ mice anterior chamber angle un-injected (left) or injected (right) with *COCH-GFP* transgene bearing lentiviral vector demonstrating an open iridocorneal angle in either case. Scale bar, 50 µm.
(TIF)

Figure S2 **Increased optic nerve damage in DBA/2J cochlin^+/+^ mice as a consequence of elevated IOP.** (A–D) Paraphenylenediamine (PPD) stained optic nerve sections (20X) show axonal death in mice. A representative PPD stained optic nerve section of 12 month old (A) DBA/2J cochlin^+/+^ (B) DBA/2J cochlin^+/−^ (C), DBA/2J cochlin^−/−^ (D) and DBA/2J-Gpnmb^+^/SjJ (E) frequency of optic nerve damage in mice at 12 months of age. Comparison of percentage of mice showing mild, moderate or severe optic nerve damage among the different strains of mice as indicated.
(EPS)

Figure S3 **Cochlin multimerization in response to fluid shear.** (A) Comparison of percentage of cochlin showing multimerization when subjected to different levels of fluid shear as indicated. The purified recombinant cochlin was subjected to different flow rates 2.5–5.0 µl/min for 50–200 cycles as indicated. Densitometric scan from four independent experiments were used to calculate mean± standard deviation, which has been presented here. (B) A representative Western blot analyses of control [not subjected to shear stress (*); as indicated] and cochlin subjected to shear stress as indicated. The purified cochlin was subjected to flow rates of 2.5–5.0 µl/min as indicated for 50–200 cycles as indicated.
(EPS)

## References

[1] Kung C (2005). A possible unifying principle for mechanosensation.. Nature.

[2] Robertson NG, Jones SM, Sivakumaran TA, Giersch AB, Jurado SA (2008). A targeted Coch missense mutation: a knock-in mouse model for DFNA9 late-onset hearing loss and vestibular dysfunction.. Hum Mol Genet.

[3] Picciani R, Desai K, Guduric-Fuchs J, Cogliati T, Morton CC (2007). Cochlin in the eye: functional implications.. Prog Retin Eye Res.

[4] Anderson DR (2003). Collaborative normal tension glaucoma study.. Curr Opin Ophthalmol.

[5] Asrani S, Zeimer R, Wilensky J, Gieser D, Vitale S (2000). Large diurnal fluctuations in intraocular pressure are an independent risk factor in patients with glaucoma.. J Glaucoma.

[6] Chow J, Liton PB, Luna C, Wong F, Gonzalez P (2007). Effect of cellular senescence on the P2Y-receptor mediated calcium response in trabecular meshwork cells.. Mol Vis.

[7] Morrison JC, Acott TS, Morrison JC, Pollack IP (2003). Anatomy and physiology of aqueous humor outflow.. Glaucoma - Science and Practice.

[8] Bhattacharya SK, Rockwood EJ, Smith SD, Bonilha VL, Crabb JS (2005). Proteomics reveals cochlin deposits associated with glaucomatous trabecular meshwork.. J Biol Chem.

[9] Patel AJ, Lazdunski M, Honore E (2001). Lipid and mechano-gated 2P domain K(+) channels.. Curr Opin Cell Biol.

[10] Goel M, Sienkiewicz AE, Picciani R, Lee RK, Bhattacharya SK (2011). Cochlin Induced TREK-1 Co-Expression and Annexin A2 Secretion: Role in Trabecular Meshwork Cell Elongation and Motility.. PLoS One.

[11] Gabelt BT, Kaufman PL (2005). Changes in aqueous humor dynamics with age and glaucoma.. Prog Retin Eye Res.

[12] John SW, Hagaman JR, MacTaggart TE, Peng L, Smithes O (1997). Intraocular pressure in inbred mouse strains.. Invest Ophthalmol Vis Sci.

[13] Wang J, Wang MR, Jiang H, Shen M, Cui L (2010). Detection of magnetic particles in live DBA/2J mouse eyes using magnetomotive optical coherence tomography.. Eye and Contact Lens: Science and Clinical Practice.

[14] Rodriguez CI, Cheng JG, Liu L, Stewart CL (2004). Cochlin, a secreted von Willebrand factor type a domain-containing factor, is regulated by leukemia inhibitory factor in the uterus at the time of embryo implantation.. Endocrinology.

[15] Libby RT, Anderson MG, Pang IH, Robinson ZH, Savinova OV (2005). Inherited glaucoma in DBA/2J mice: pertinent disease features for studying the neurodegeneration.. Vis Neurosci.

[16] Bhattacharya SK, Gabelt BT, Ruiz J, Picciani R, Kaufman PL (2009). Cochlin expression in anterior segment organ culture models after TGFbeta2 treatment.. Invest Ophthalmol Vis Sci.

[17] Lee ES, Gabelt BT, Faralli JA, Peters DM, Brandt CR (2010). COCH transgene expression in cultured human trabecular meshwork cells and its effect on outflow facility in monkey organ cultured anterior segments.. Invest Ophthalmol Vis Sci.

[18] Jimenez AI, Sesto A, Roman JP, Gascon I, Gonzalez de Buitrago G, Office USPaT, editor (2007). Treatment of eye disorders characterized by an elevated intraocular pressure by siRNAS.. US Patent.

[19] Heys JJ, Barocas VH, Taravella MJ (2001). Modeling passive mechanical interaction between aqueous humor and iris.. J Biomech Eng.

[20] Vass CH C, Unger E, Mayr W, Georgopoulos M, Rainer G, Richter-Mueksch S (2004). Human aqueous humor viscosity in cataract, primary open angle glaucoma and pseudoexfoliation syndrome.. Invest Ophthalmol Vis Sci E Abstract.

[21] Sit AJ, Nau CB, McLaren JW, Johnson DH, Hodge D (2008). Circadian variation of aqueous dynamics in young healthy adults.. Invest Ophthalmol Vis Sci.

[22] Larsson LI, Rettig ES, Brubaker RF (1995). Aqueous flow in open-angle glaucoma.. Arch Ophthalmol.

[23] Shankaran H, Alexandridis P, Neelamegham S (2003). Aspects of hydrodynamic shear regulating shear-induced platelet activation and self-association of von Willebrand factor in suspension.. Blood.

[24] Bhattacharya SK, Annangudi SP, Salomon RG, Kuchtey RW, Peachey NS (2005). Cochlin deposits in the trabecular meshwork of the glaucomatous DBA/2J mouse.. Exp Eye Res.

[25] Vogel V (2006). Mechanotransduction involving multimodular proteins: converting force into biochemical signals.. Annu Rev Biophys Biomol Struct.

[26] WuDunn D (2009). Mechanobiology of trabecular meshwork cells.. Exp Eye Res.

[27] Geiger B, Bershadsky A, Pankov R, Yamada KM (2001). Transmembrane crosstalk between the extracellular matrix–cytoskeleton crosstalk.. Nat Rev Mol Cell Biol.

[28] Janmey PA, Weitz DA (2004). Dealing with mechanics: mechanisms of force transduction in cells.. Trends Biochem Sci.

[29] Fujiwara K (2006). Platelet endothelial cell adhesion molecule-1 and mechanotransduction in vascular endothelial cells.. J Intern Med.

[30] Borras T (2003). Gene expression in the trabecular meshwork and the influence of intraocular pressure.. Prog Retin Eye Res.

[31] Kommareddi PK, Nair TS, Raphael Y, Telian SA, Kim AH (2007). Cochlin isoforms and their interaction with CTL2 (SLC44A2) in the inner ear.. J Assoc Res Otolaryngol.

[32] Lesage F, Lazdunski M (2000). Molecular and functional properties of two-pore-domain potassium channels.. Am J Physiol Renal Physiol.

[33] Lauritzen I, Chemin J, Honore E, Jodar M, Guy N (2005). Cross-talk between the mechano-gated K2P channel TREK-1 and the actin cytoskeleton.. EMBO Rep.

[34] Chemin J, Patel AJ, Duprat F, Lauritzen I, Lazdunski M (2005). A phospholipid sensor controls mechanogating of the K+ channel TREK-1.. Embo J.

[35] Sharif-Naeini R, Folgering JH, Bichet D, Duprat F, Lauritzen I (2009). Polycystin-1 and -2 dosage regulates pressure sensing.. Cell.

[36] Lambrechts A, Jonckheere V, Peleman C, Polet D, De Vos W (2006). Profilin-I-ligand interactions influence various aspects of neuronal differentiation.. J Cell Sci.

[37] Syriani E, Gomez-Cabrero A, Bosch M, Moya A, Abad E (2008). Profilin induces lamellipodia by growth factor-independent mechanism.. Faseb J.

[38] Morales M, Gomez-Cabrero A, Peral A, Gasull X, Pintor J (2007). Hypotensive effect of profilin on rabbit intraocular pressure.. Eur J Pharmacol.

[39] Gomez-Cabrero A, Comes N, Gonzalez-Linares J, de Lapuente J, Borras M (2005). Use of transduction proteins to target trabecular meshwork cells: outflow modulation by profilin I.. Mol Vis.

[40] Maepea O, Bill A (1992). Pressures in the juxtacanalicular tissue and Schlemm's canal in monkeys.. Exp Eye Res.

[41] Tian B, Gabelt BT, Geiger B, Kaufman PL (2009). The role of the actomyosin system in regulating trabecular fluid outflow.. Exp Eye Res.

[42] Zhang Y, Toris CB, Liu Y, Ye W, Gong H (2009). Morphological and hydrodynamic correlates in monkey eyes with laser induced glaucoma.. Exp Eye Res.

[43] Gong H, Freddo TF (2009). The washout phenomenon in aqueous outflow–why does it matter?. Exp Eye Res.

[44] Quigley HA, Broman AT (2006). The number of people with glaucoma worldwide in 2010 and 2020.. Br J Ophthalmol.

[45] Morrison JC, Johnson EC, Cepurna W, Jia L (2005). Understanding mechanisms of pressure-induced optic nerve damage.. Prog Retin Eye Res.

