# Supplementary figures and images for: Cochlin, Intraocular Pressure Regulation and Mechanosensing
Source: PLoS One. 2012 Apr 4;7(4):e34309. doi: 10.1371/journal.pone.0034309 (PMC3319572; doi:10.1371/journal.pone.0034309)

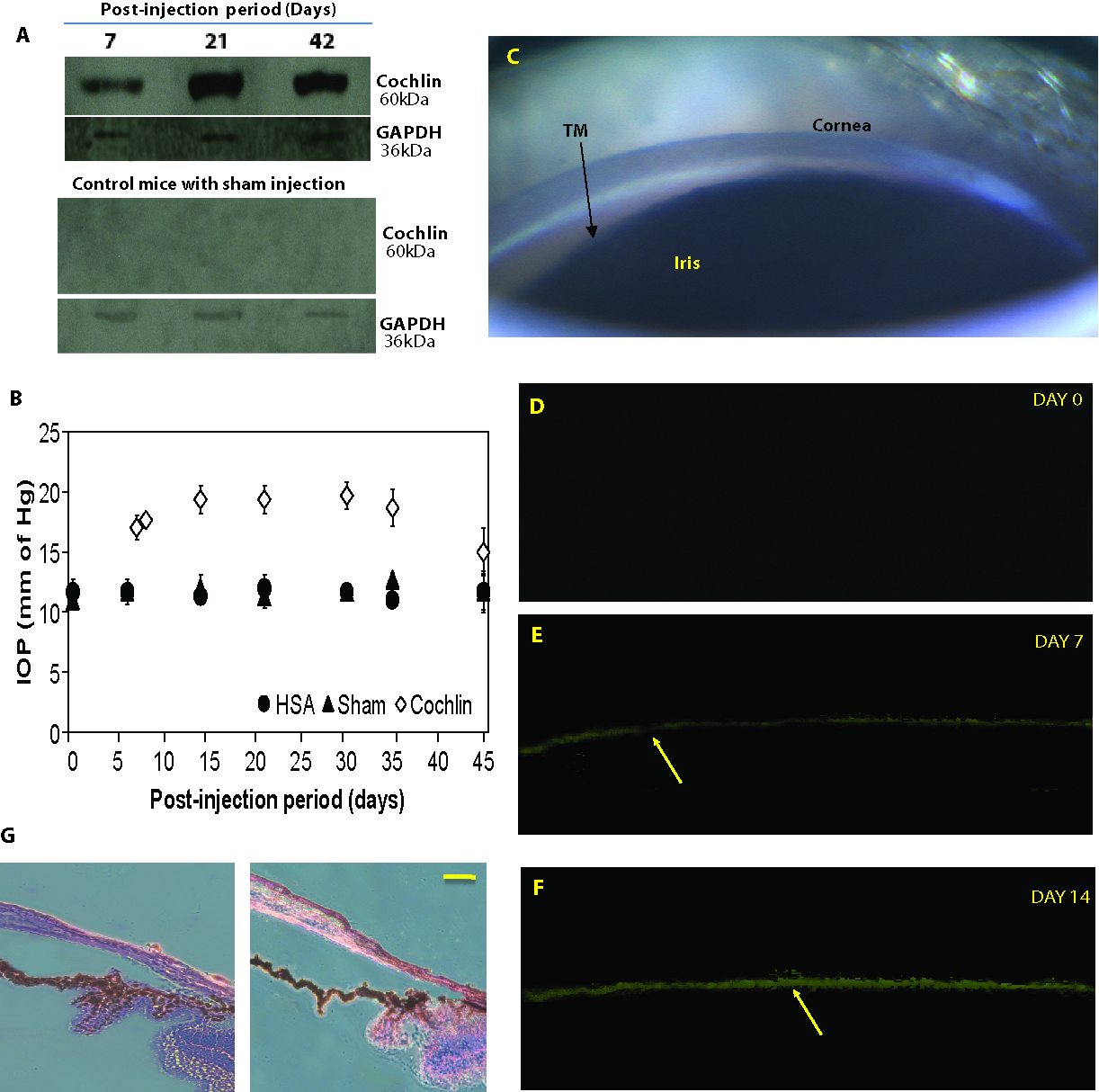

Supplement: Figure S1 — COCH-GFP transgene injection leads to cochlin expression in-vivo. (A) Western analysis of the TM protein extract (10 µg) of the DBA/2J-Gpnmb+/SjJ mice injected with COCH transgene on days 7, 21 and 42 post-injection. GAPDH has been shown as a loading control. The lower panels indicate Western blot from control animals as indicated. (B) DBA/2J-Gpnmb+/SjJ mice (n = 45) at six months were injected (1 µl) with 10 µg of purified human serum albumin (HSA), Cochlin or PBS (sham) in the anterior chamber as indicated every 72 hours up to 44 days. (C) Image of a living mouse anterior chamber angle showing the cornea, iris and the TM (arrow). (D–F) In vivo imaging of GFP expression in mice TM. Before COCH-GFP injection [DAY 0, (D)] no fluorescence is seen in the TM but at DAY 7 (E) and DAY 14 (F) post-injection, GFP expression (arrow) can be visualized. (G) Hematoxylin-eosin stained images of DBA/2J-Gpnmb+/SjJ mice anterior chamber angle un-injected (left) or injected (right) with COCH-GFP transgene bearing lentiviral vector demonstrating an open iridocorneal angle in either case. Scale bar, 50 µm. (TIF) [file pone.0034309.s001.tif]
